# Usefulness of the Optimal Cutoff Value and Delta Value of Leucine-Rich Alpha 2 Glycoprotein in Ulcerative Colitis

**DOI:** 10.1093/crocol/otac039

**Published:** 2022-11-03

**Authors:** Satohiro Matsumoto, Hirosato Mashima

**Affiliations:** Department of Gastroenterology, Jichi Medical University Saitama Medical Center, Saitama, Saitama, Japan; Department of Gastroenterology, Jichi Medical University Saitama Medical Center, Saitama, Saitama, Japan

**Keywords:** leucine-rich alpha 2 glycoprotein, cutoff value, delta value, mucosal healing, ulcerative colitis

## Abstract

**Background:**

Leucine-rich alpha 2 glycoprotein (LRG) is a novel serum biomarker used to determine disease activity in inflammatory bowel disease. We investigated the association between endoscopic scores based on the Ulcerative Colitis Endoscopic Index of Severity (UCEIS) and LRG in ulcerative colitis (UC).

**Methods:**

A total of 1019 LRG measurements were obtained from 358 patients with UC. This study included 190 patients (199 measurements) who underwent colonoscopy within 3 months before and after LRG measurement with unchanged disease status or treatment during the same period. The patients were divided into those with and without UC relapse. We evaluated the correlation between LRG levels and UCEIS scores and performed a receiver operating characteristic curve analysis to determine the optimal LRG cutoff value. Delta values of LRG were then analyzed.

**Results:**

LRG levels were positively correlated with UCEIS scores (correlation coefficient: 0.638; 95% CI: 0.548–0.714; *P* < .0001) in all disease types. The LRG cutoff value for mucosal healing was 12.6 µg mL^−1^ (area under the curve: 0.736; 95% CI: 0.651–0.821); this value had a sensitivity of 0.72 and a specificity of 0.66. In patients with UC relapse, the median delta value of LRG before and after relapse was 5 µg mL^−1^.

**Conclusions:**

LRG levels were positively correlated with the UCEIS scores. The optimal LRG cutoff value for determining mucosal healing was 12.6 µg mL^−1^. The median delta value of LRG before and after relapse was 5 µg mL^−1^.

## Introduction

Ulcerative colitis (UC) is a type of chronic inflammatory bowel disease (IBD) of the colon that has an unknown cause. In Japan, the number of patients diagnosed with UC has been increasing every year; registered patients reached 200 000 in 2016. UC has been treated with biological agents such as anti-tumor necrosis factor (TNF)-α agents, anti-interleukin (IL) 12/23 antibodies, and anti-α4β7 integrin antibodies, and Janus kinase (JAK) inhibitors (the molecular targeted drug). Although the number of treatment options has increased, no definitive treatment has been established. Hence, regular control of disease activity and long-term remission maintenance therapy are necessary.

The objective of drug efficacy evaluation is endoscopic mucosal and histological healing. To achieve endoscopic mucosal healing, disease activity must be regularly controlled and maintained at low levels by monitoring clinical symptoms (eg, diarrhea, bloody stools, and abdominal pain) and laboratory values (eg, C-reactive protein [CRP]) in daily clinical practice. CRP is an acute-phase protein that is synthesized in the liver after stimulation with IL-6.^1^ It is the most widely used surrogate marker for monitoring the clinical disease activity of IBD.^2^ However, the existing serum markers represented by CRP are not sensitive enough to identify all patients with endoscopic disease activity because some patients with endoscopically proven intestinal tract inflammation have mild clinical symptoms or normal CRP levels, making it difficult to evaluate disease activity.

To identify novel proteins involved in inflammation, Serada et al performed semiquantitative protein analysis on serum samples collected from patients with rheumatoid arthritis before and after treatment with anti-TNF-α antibodies and identified leucine-rich alpha 2 glycoprotein (LRG), a novel acute-phase protein.^3^ Subsequently, LRG was confirmed to be useful for evaluating the disease activity of IBD and was covered by the National Health Insurance System in Japan in June 2020. LRG levels are correlated with endoscopic disease activity and are considered useful for evaluating disease activity even in patients with normal CRP levels.^4,5^

Although the correlation between LRG levels and endoscopic disease activity of UC has been comprehensively analyzed using the Mayo endoscopic score (MES), there have been no reports of such analysis using the Ulcerative Colitis Endoscopic Index of Severity (UCEIS) to date. Here, we analyzed LRG levels and endoscopic disease activity in patients with UC to examine whether LRG can be a potential surrogate marker for mucosal healing and investigate the clinical significance of LRG.

## Materials and Methods

### Subjects

This retrospective single-center study was conducted at Jichi Medical University Saitama Medical Center. From the medical records, 358 Japanese patients with UC who visited Saitama Medical Center between July 2020 and November 2021 and had serum LRG levels measured at least once were selected and enrolled in this study. Patients who had an obvious infection at the time of blood collection for LRG measurement and those who received SARS-CoV-2 vaccination within 1 week before blood collection were excluded. A total of LRG measurements was obtained from 358 patients. Nanopia LRG (Sekisui Medical) was used for LRG measurements. First, the association between LRG levels and clinical disease activity was analyzed. Of the 358 patients, 250 underwent colonoscopy during the study period. Among the 250 patients, 190 patients (199 measurements) who underwent colonoscopy within 3 months before and after LRG measurement and experienced no changes in either disease status or treatment during the same period were included in the analysis. The association between LRG levels and endoscopic disease activity was analyzed to determine the optimal cutoff value for predicting mucosal healing. While the normal CRP level was set at <2 mg L^−1^, the association between LRG levels and endoscopic disease activity was analyzed in CRP-negative patients. To verify the usefulness of LRG in real-world clinical practice, changes in LRG levels were examined in patients who experienced UC relapse during the observation period. Furthermore, the delta values of LRG were analyzed, and the patients were divided into those with and without UC relapse.

### Assessment of Clinical and Endoscopic Disease Activity

Clinical symptoms were scored using the clinical activity index (CAI) developed by Lichtiger et al.^6^ The components of the CAI were as follows: bowel movement frequency (score, 0–4), nocturnal diarrhea (score, 0–1), blood in the stool (score, 0–3), fecal incontinence (score, 0–1), use of antidiarrheal drugs (score, 0–1), abdominal pain (score, 0–3), general well-being (score, 0–5), and abdominal tenderness (score, 0–3). Clinical remission was defined as CAI ≤3.^6^ Endoscopic findings were quantified using the UCEIS developed by Travis et al.^7^ The 3 UCEIS scores were as follows: vascular pattern (score, 0–2), bleeding (score, 0–3), and erosion/ulceration (score, 0–3). The sum of the scores for each endoscopic variable ranged from 0 to 8. The scores for each patient were determined by assessing areas with the most severe inflammation. Mucosal healing was defined as a UCEIS score of 1 or less.

### Ethical Considerations

This study was approved by the Etiological Study Ethical Review Board of Jichi Medical University Saitama Medical Center. Because we produced and used anonymized data, informed consent from the participants was not required.

### Statistical Analyses

Data are expressed as mean ± standard deviation or percentage. LRG levels were compared using Student’s *t*-test. Spearman’s rank correlation coefficient was used to measure the strength and direction of correlation between the 2 variables. The receiver operating characteristic (ROC) curve is a plot of the true positive fraction vs. the false positive fraction, and the area under the ROC curve (AUC) was calculated to determine the optimal cutoff value. All statistical analyses were performed using EZR (The R Foundation for Statistical Computing, version 1.54).^8^ Differences were regarded as significant at *P* values <.05.

## Results

### LRG Concentration in Relation to Clinical Activity

**Figure 1.**
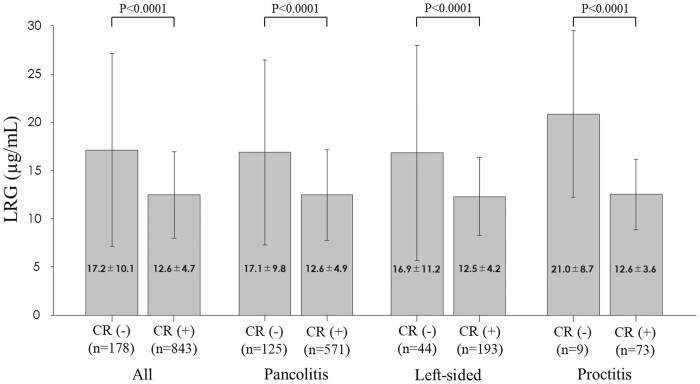
Serum LRG levels in patients with UC categorized according to disease activity and disease location. Abbreviations: LRG, leucine-rich alpha 2 glycoprotein; UC, ulcerative colitis.

Table 1 shows the patient characteristics. The mean age of the patients was 46 ± 16 years (15–88 years). The disease types included pancolitis (66.8%), left-sided colitis (22.6%), and proctitis (10.1%). The measurement interval between colonoscopy and LRG was 54.4 ± 29.1 (0–90) days. A strong positive correlation was observed between the LRG and CRP levels (correlation coefficient: 0.849; 95% CI: 0.806–0.883; *P* < .0001). When LRG levels were compared between patients with UC in clinical remission and those with clinically active UC, serum LRG levels were higher in patients with clinically active UC than in those in clinical remission, which was also true when compared with those in clinical remission for all disease types (Figure 1). A positive correlation was observed between serum LRG levels and CAI scores (correlation coefficient: 0.453; 95% CI: 0.403–0.501; *P* < .0001).

**Table 1.** Baseline characteristics.

	All patients (*n* = 358)	Subjects (*n* = 190)
Male, number	195 (54.5%)	108 (56.8%)
Age at onset, y	35 ± 15 (3–82)	35 ± 15 (11–75)
Age at entry, y	46 ± 16 (15–88)	46 ± 16 (16–81)
Extent disease		
Pancolitis	239 (66.8%)	136 (71.6%)
Left-sided colitis	81 (22.6%)	39 (20.5%)
Proctitis	36 (10.1%)	14 (7.4%)
Right-sided colitis	2 (0.6%)	1 (0.5%)
Duration of ulcerative colitis, y	10.5 ± 8.8 (0–51.0)	10.5 ± 8.8 (0–51.0)
Previous treatment		
Cytapheresis	56 (15.6%)	41 (21.6%)
Tacrolimus	29 (8.1%)	24 (12.6%)
Steroid	217 (60.6%)	127 (66.8%)
Treatment at entry		
Mesalazine/salazosulfapyridine	352 (98.3%)	185 (97.4%)
Immunomodulators	137 (38.3%)	86 (45.3%)
Biologics/JAK inhibitor	119 (33.2%)	77 (40.5%)
Infliximab	11 (3.1%)	9 (4.7%)
Adalimumab	21 (5.9%)	14 (7.4%)
Golimumab	10 (2.8%)	7 (3.7%)
Vedolizumab	33 (9.2%)	22 (11.6%)
Ustekinumab	18 (5.0%)	10 (5.3%)
Tofacitinib	15 (4.2%)	15 (7.9%)
CAI	3.4 ± 1.4 (2–13)	3.5 ± 1.6 (2–13)
LRG (µg mL^−1^)	13.3 ± 6.1 (5–68.5)	13.2 ± 6.6 (5–56.7)
C-reactive protein (mg L^−1^)	2.9 ± 14.4 (0.1–334.1)	2.6 ± 6.7 (0.1–47.8)
UCEIS		1.2 ± 1.7 (0–7)

Abbreviations: CAI, clinical activity index; JAK, Janus kinase; LRG, leucine-rich alpha 2 glycoprotein; UCEIS, Ulcerative Colitis Endoscopic Index of Severity.

### LRG Concentration in Relation to Endoscopic Activity

**Figure 2.**
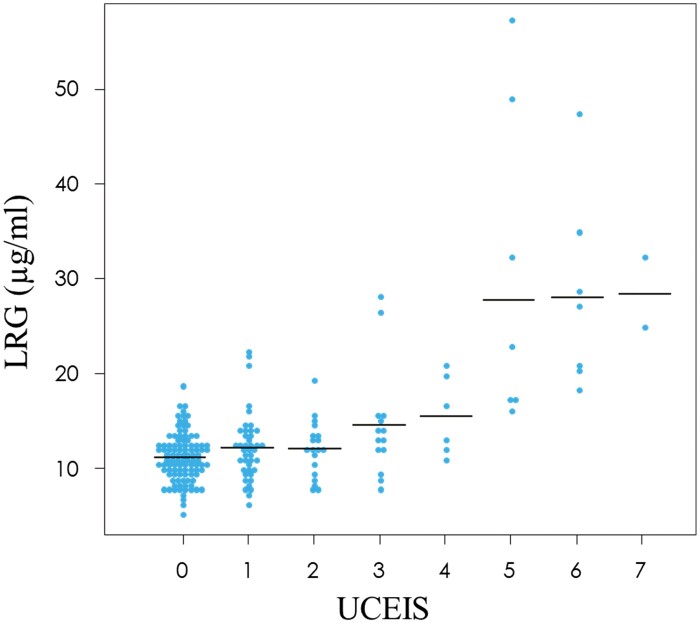
Serum LRG levels plotted according to endoscopic activity in patients with UC. Abbreviations: LRG, leucine-rich alpha 2 glycoprotein; UC, ulcerative colitis.

**Figure 3.**
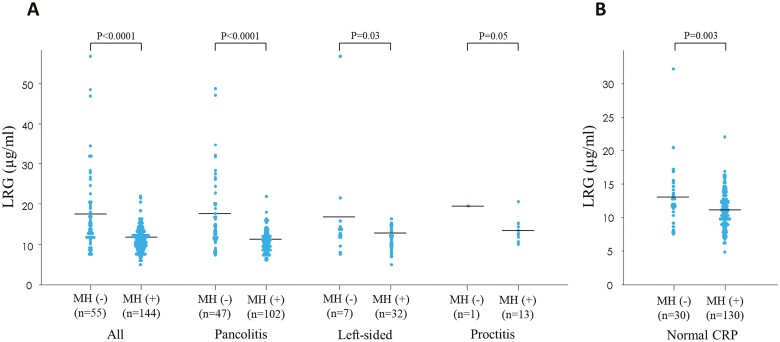
Serum LRG levels between the MH group and non-MH group in patients with UC categorized according to disease location (A). Serum LRG levels between the MH group and non-MH group in patients with UC with normal CRP levels (<2 mg L^−1^) (B). Abbreviations: CRP, C-reactive protein; LRG, leucine-rich alpha 2 glycoprotein; MH, mucosal healing; UC, ulcerative colitis.

We then evaluated whether LRG levels are associated with endoscopic disease activity in patients with UC. Since LRG levels steadily increased when UCEIS scores increased, the severity of colonic mucosal inflammation was significantly correlated with the increase in LRG levels (correlation coefficient: 0.638; 95% CI: 0.548–0.714; *P* < .0001) (Figure 2). Additionally, significantly positive correlations were observed between serum LRG levels and UCEIS scores in all disease types: pancolitis (correlation coefficient: 0.684; 95% CI: 0.587–0.762; *P* < .0001), left-sided colitis (correlation coefficient: 0.597; 95% CI: 0.342–0.770; *P* < .0001), and proctitis (correlation coefficient: 0.54; 95% CI: 0.013–0.832; *P* = .0464). LRG levels were significantly higher in patients without mucosal healing than in those with mucosal healing (Figure 3A). Similar results were observed for pancolitis and left-sided colitis according to the disease type (Figure 3A). Even among patients with UC who showed normal CRP levels (<2 mg L^−1^), LRG levels in patients who did not achieve mucosal healing were significantly higher than in those who achieved mucosal healing (Figure 3B).

### Cutoff Value of LRG for Evaluation of Mucosal Healing in Patients With UC

**Figure 4.**
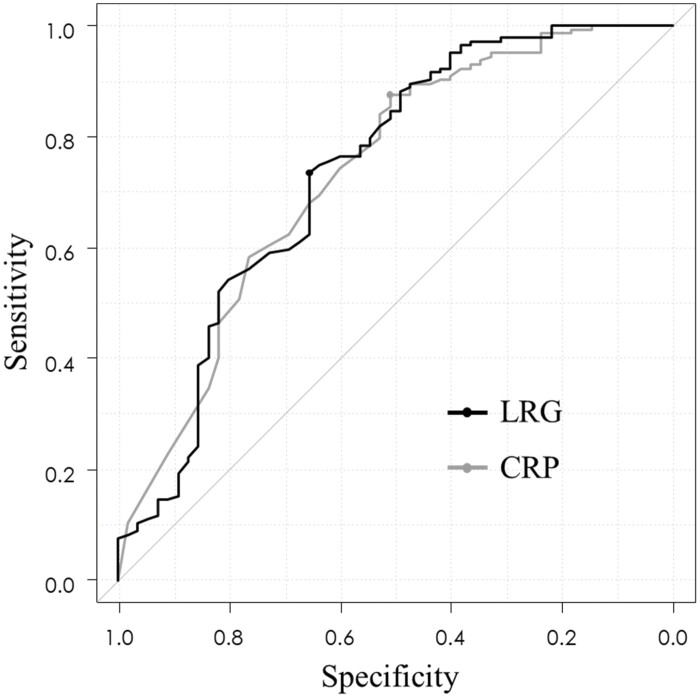
The receiver operating characteristic curve of LRG for predicting mucosal healing. Abbreviation: LRG, leucine-rich alpha 2 glycoprotein.

Figure 4 shows an ROC curve demonstrating the ability of LRG and CRP to detect mucosal healing. For mucosal healing, the AUC for LRG was 0.736 (95% CI: 0.651–0.821) with a cutoff value of 12.6 µg mL^−1^. The AUC for CRP was 0.733 (95% CI: 0.650–0.815) and similar with that of LRG. Thus, the optimal LRG cutoff value for evaluating mucosal healing was 12.6 µg mL^−1^. Regarding diagnostic accuracy, the LRG cutoff value for mucosal healing had sensitivities of 0.72, specificities of 0.66, positive predictive values of 0.84, and negative predictive values of 0.47.

### Monitoring the Clinical Course of Patients With UC Using LRG

During the observation period, relapse occurred in 23 patients (6.4%), of whom 8 (34.8%) had elevated LRG levels before relapse. In 7 of the 8 patients, serum LRG levels before relapse were ≥12.6 µg mL^−1^, and relapse occurred within 1 month after the last LRG measurement. In the remaining patient, although the serum LRG level before relapse was 11.7 µg mL^−1^, which was lower than 12.6 µg mL^−1^, the LRG level 3 months earlier was 7.7 µg mL^−1^. Three months after relapse, clinical remission was achieved, and the LRG level decreased to 6.7 µg mL^−1^. When the patients were divided into those with relapse (23 patients) and those without relapse (335 patients) and analyzed, the mean LRG levels at the prior measurement point were 13.7 ± 5.7 (8–32) and 12.5 ± 4.2 (5–42.6) µg mL^−1^ in patients with and without relapse, respectively, which were not significantly different (*P* = .27). Meanwhile, the mean LRG delta values were 8.5 ± 16.0 (−9.4 to 56.1) and −0.8 ± 4.3 (−44.8 to 17.9) µg mL^−1^ in patients with and without relapse, respectively. The mean delta value was significantly higher in patients who relapsed (*P* < .0001) (Table 2). The median delta value in patients with relapse was 5 µg mL^−1^.

**Table 2.** The serum LRG level between patients with and without relapse.

	Relapse (*n* = 23)	Non-relapse (*n* = 335)	*P*
LRG at the prior measurement point (µg mL^−1^)			
All	13.7 ± 5.7 (8 to 32)	12.5 ± 4.2 (5 to 42.6)	.27
Pancolitis	14.0 ± 6.6 (8 to 32)	12.2 ± 4.0 (5.9 to 39.1)	.13
Left-sided colitis	12.7 ± 3.1 (8.9 to 16.2)	12.6 ± 3.9 (5 to 26.6)	.97
Proctitis	NA	14.5 ± 5.9 (6.8 to 42.6)	
LRG delta (µg mL^−1^)			
All	8.5 ± 16.0 (−9.4 to 56.1)	−0.8 ± 4.3 (−44.8 to 17.9)	<.0001
Pancolitis	9.2 ± 17.6 (8 to 32)	−0.9 ± 4.0 (−37.2 to 9.4)	<.0001
Left-sided colitis	6.9 ± 13.0 (−4.8 to 29.1)	−0.8 ± 4.9 (−44.8 to 8.8)	.002
Proctitis	NA	−0.1 ± 5.0 (−23 to 17.9)	

Abbreviation: LRG, leucine-rich alpha 2 glycoprotein.

### Cases With Negative LRG and Positive CRP

Although LRG is an excellent marker that reflects colonic mucosal inflammation, there are patients who cannot be identified even by the cutoff or delta values of LRG. We defined LRG negativity as an LRG level of <12.6 µg mL^−1^ and CRP positivity as a CRP level of ≥2 mg L^−1^. Supplementary Table 1 shows the 11 (5.5%) patients who were LRG negative and CRP positive. The highest CRP level among these patients was 4.3 mg L^−1^. There were no LRG-negative patients with CRP levels >5 mg L^−1^. In 36.3% (4/11) of these patients, the UCEIS score was 2 or higher, indicating endoscopically active UC. In contrast, 52 patients were LRG positive and CRP negative, accounting for 26.1% (52/199). In 26.9% (14/52) of these patients, the UCEIS score was 2 or higher.

## Discussion

A meta-analysis reported that mucosal healing reduces the risks of recurrence and surgery and maintains long-term mucosal healing.^9^ As mucosal healing as a treatment goal has been widely accepted, methods to evaluate mucosal healing have attracted attention. Colonoscopy is essential for evaluating mucosal healing; however, it is invasive and difficult to perform frequently. Moreover, it may aggravate symptoms in patients with UC.^10^ Thus, there is a need for reliable noninvasive surrogate markers that can be used as substitutes for colonoscopy. Among surrogate markers for evaluating mucosal inflammation, both fecal calprotectin (Fcal) and fecal immunochemical test (FIT) are significantly correlated with endoscopic disease activity.^11–14^ Fcal is useful in predicting relapse in patients with UC.^15–18^ However, fecal markers are associated with several problems, including inadequate collection of fecal samples. As for problems specific to Fcal, its levels may be elevated even in diseases other than IBD, and its disease specificity is low. The Fcal test can also yield positive results in patients with colorectal cancer, and Fcal levels are affected by age, obesity, and oral non-steroidal anti-inflammatory drugs. Moreover, multiple test kits are available, but no cutoff values have been established.^19^ As for FIT, it may yield positive results for fecal samples collected during hemorrhoidal bleeding and menstruation. FIT may also yield false-positive results in patients with multiple large inflammatory polyps even if no inflammation is detected by endoscopy.

Since there are no defined cutoff values for Fcal, most studies have used cutoff values based on ROC curves. On the other hand, although the reference value of LRG is 16.0 µg mL^−1^, reports on cutoff values for predicting mucosal healing in UC are extremely limited. Yasutomi et al have reported that the LRG cutoff value for complete mucosal healing, defined as an MES of 0, in patients with UC was 12.7 µg mL^−1^.^20^ Although the present study identified an LRG level of 12.6 µg mL^−1^ as the cutoff value for mucosal healing, the specificity was as low as 66%. The present study revealed that baseline LRG levels, which were suggestive of mucosal healing, differed among patients.

We consider LRG delta values as more important than the cutoff values for evaluating mucosal healing. Shinzaki et al analyzed the association between the rate of decrease in LRG levels and mucosal healing in UC patients. According to their analysis, none of the patients in whom LRG levels decreased by ≥30% showed endoscopic aggravation. In contrast, 71.4% of the patients in whom LRG levels decreased by <30% showed endoscopic aggravation. These results suggest that LRG is a useful tool for monitoring the mucosal status of patients with UC.^4^ In the present study, 35% of patients who experienced relapse during the observation period had elevated LRG levels before relapse; hence, LRG might be useful for predicting relapse. Patients with an LRG level of <12.6 µg mL^−1^ and a CRP level of ≥ 2 mg L^−1^ accounted for only 5.5% of all patients; 36.3% of these had a UCEIS score of ≥2, which indicated that UC was endoscopically active. In contrast, patients with an LRG level of ≥12.6 µg mL^−1^ and a CRP level <2 mg L^−1^ accounted for 26.1% of all patients. Despite a strong positive correlation between LRG and CRP levels, monitoring of CRP levels alone is insufficient, and mucosal inflammation is not detected in some patients by monitoring LRG cutoff values alone. These results suggest that attention should be paid to the delta values of the LRG.

Although the use of noninvasive biomarkers that can be repeatedly performed is important in daily clinical practice, endoscopy remains the gold standard for evaluating disease activity in UC. Several scoring systems have been used to evaluate endoscopic disease activity in UC. Among these, MES is the most representative,^21^ and UCEIS more accurately reflects clinical outcomes and long-term prognosis.^22,23^ Ikeya et al have reported that UCEIS more accurately reflects clinical outcomes and long-term prognosis than MES.^22^ Although mucosal healing has been defined as an MES of 0 or 1, the relapse rate has been reported to be higher in patients with an MES of 1 than in those with an MES of 0. In recent years, mucosal healing has been defined as an MES of 0.^24,25^ Vuitton et al reported that most of the 15 IBD specialists agreed to define endoscopic remission as a UCEIS score of 0.^26^ However, the cutoff value of UCEIS for mucosal healing has not yet been determined. Therefore, further investigation is required. In the present study, we defined mucosal healing as a UCEIS score of 0 or 1 and analyzed the data. However, the extent of lesions is not reflected in either the MES or UCEIS scores. To obtain more accurate data, we consider it necessary to develop modified UCEIS scores that reflect the extent of the lesions. For instance, UCEIS scores are calculated for individual sites, such as the cecum/ascending colon, transverse colon, descending colon, sigmoid colon, and rectum according to the current standard procedure, and the scores are then summed.

One of the difficulties in treating UC is asymptomatic but active inflammation. Colonoscopy revealed active inflammation in 45% of asymptomatic patients whose CRP levels, white blood cell counts, and erythrocyte sedimentation rates were all within normal range.^27^ In the present study, endoscopically active inflammation, defined as a UCEIS score of ≥2, was detected in 18% (30/163) of the patients in clinical remission (CAI ≤3) (Supplementary Figure 1A). When the analysis was restricted to 57 patients with a UCEIS score of ≥2, 58% of the 33 patients in clinical remission had an LRG level of ≥12.6 µg mL^−1^ (Supplementary Figure 1B). When the analysis was further restricted to CRP-negative patients, 45% of the 22 patients in clinical remission had an LRG level of ≥12.6 µg mL^−1^ (Supplementary Figure 1C). In other words, approximately 20% of patients, even those in clinical remission, had endoscopically active inflammation. These patients showed elevated LRG levels, and CRP-negative patients showed similarly elevated LRG levels. These findings suggest that LRG is useful for identifying patients with asymptomatic active inflammation and is effective even in CRP-negative patients.

How should LRG be used? We would like to propose that after remission induction therapy, patients should be treated until a target LRG level of <12.6 µg mL^−1^ is reached and then monitored for remission maintenance by evaluating delta values from the baseline LRG level, which is individually set as the LRG level at the time of achieving mucosal healing. Although further accumulation of cases is necessary with respect to the delta values, an increase from baseline by 5 µg mL^−1^ or more suggests a relapse of UC.

The present study has several limitations. This was a retrospective study conducted at a single center. LRG measurements and endoscopy were performed on different days in most patients. Because blood samples were rarely collected from patients with proctitis or mild UC, LRG measurements were unavailable in many patients with these conditions. Thus, the present study was affected by selection bias. However, we regard the present study as valuable because we analyzed >1000 LRG measurements.

## Conclusion

LRG levels were positively correlated with UCEIS scores. The optimal LRG cutoff value for determining mucosal healing is 12.6 µg mL^−1^. However, because this value is associated with low specificity and a high false-positive rate, we consider LRG delta values as also being useful for monitoring in addition to the cutoff value.

## Supplementary Data

Supplementary data is available at *Crohn’s and Colitis 360* online.

Supplementary Figure 1. UCEIS score plotted according to clinical activity in patients with UC (A). Serum LRG levels plotted according to clinical activity in patients with UC (B). Serum LRG levels plotted according to clinical activity with normal CRP levels (<2 mg L^−1^) (C).

otac039_suppl_Supplementary_Figure_S1

otac039_suppl_Supplementary_Table_S1

otac039_suppl_Supplementary_Figure_Legend

## Data Availability

Data not publicly available.
