# Supplementary material for: Usefulness of the Optimal Cutoff Value and Delta Value of Leucine-Rich Alpha 2 Glycoprotein in Ulcerative Colitis
Source: Crohns Colitis 360. 2022 Nov 3;4(4):otac039. doi: 10.1093/crocol/otac039 (PMC9681229; doi:10.1093/crocol/otac039)
Supplement: otac039_suppl_Supplementary_Table_S1 [file otac039_suppl_supplementary_table_s1.docx]

| Supplementary Table 1　 11 cases with negative LRG and positive CRP | | | | | |
| --- | --- | --- | --- | --- | --- |
| **Sex** | **Extent disease** | **LRG (μg/mL)** | **CRP (mg/L)** | **UCEIS** | **CAI** |
| Male | Left-sided | 11.9 | 2.0 | 2 | 9 |
| Male | Pancolitis | 8.6 | 2.1 | 3 | 3 |
| Male | Pancolitis | 12.1 | 2.2 | 0 | 3 |
| Male | Pancolitis | 7.5 | 2.6 | 0 | 3 |
| Male | Pancolitis | 11.8 | 2.8 | 0 | 3 |
| Male | Left-sided | 11.8 | 3.0 | 0 | 4 |
| Female | Left-sided | 12 | 3.1 | 0 | 3 |
| Male | Pancolitis | 9.1 | 3.1 | 3 | 4 |
| Male | Pancolitis | 11.8 | 3.6 | 2 | 4 |
| Female | Pancolitis | 12.2 | 4.1 | 0 | 4 |
| Male | Pancolitis | 10.3 | 4.3 | 0 | 3 |
