# Supplementary material for: Usefulness of the Optimal Cutoff Value and Delta Value of Leucine-Rich Alpha 2 Glycoprotein in Ulcerative Colitis
Source: Crohns Colitis 360. 2022 Nov 3;4(4):otac039. doi: 10.1093/crocol/otac039 (PMC9681229; doi:10.1093/crocol/otac039)
Supplement: otac039_suppl_Supplementary_Figure_Legend [file otac039_suppl_supplementary_figure_legend.docx]

[Supplementary Figure]

Supplementary Fig 1. UCEIS score plotted according to clinical activity in patients with UC (A). Serum LRG levels plotted according to clinical activity in patients with UC (B). Serum LRG levels plotted according to clinical activity with normal CRP levels (< 2 mg/L) (C)
